# Identification of Subtype Specific miRNA-mRNA Functional Regulatory Modules in Matched miRNA-mRNA Expression Data: Multiple Myeloma as a Case

**DOI:** 10.1155/2015/501262

**Published:** 2015-03-19

**Authors:** Yunpeng Zhang, Wei Liu, Yanjun Xu, Chunquan Li, Yingying Wang, Haixiu Yang, Chunlong Zhang, Fei Su, Yixue Li, Xia Li

**Affiliations:** ^1^College of Bioinformatics Science and Technology, Harbin Medical University, 194 Xuefu Road, Harbin 150081, China; ^2^Department of Mathematics, Heilongjiang Institute of Technology, Harbin 150050, China

## Abstract

Identification of miRNA-mRNA modules is an important step to elucidate their combinatorial effect on the pathogenesis and mechanisms underlying complex diseases. Current identification methods primarily are based upon miRNA-target information and matched miRNA and mRNA expression profiles. However, for heterogeneous diseases, the miRNA-mRNA regulatory mechanisms may differ between subtypes, leading to differences in clinical behavior. In order to explore the pathogenesis of each subtype, it is important to identify subtype specific miRNA-mRNA modules. In this study, we integrated the Ping-Pong algorithm and multiobjective genetic algorithm to identify subtype specific miRNA-mRNA functional regulatory modules (MFRMs) through integrative analysis of three biological data sets: GO biological processes, miRNA target information, and matched miRNA and mRNA expression data. We applied our method on a heterogeneous disease, multiple myeloma (MM), to identify MM subtype specific MFRMs. The constructed miRNA-mRNA regulatory networks provide modular outlook at subtype specific miRNA-mRNA interactions. Furthermore, clustering analysis demonstrated that heterogeneous MFRMs were able to separate corresponding MM subtypes. These subtype specific MFRMs may aid in the further elucidation of the pathogenesis of each subtype and may serve to guide MM subtype diagnosis and treatment.

## 1. Introduction

MicroRNAs (miRNAs) are a class of short, noncoding RNAs of ~22 nucleotides RNA molecules that play important roles in gene regulation during physiological or disease-associated processes [1]. By regulating gene expression, miRNAs are involved in most biological processes, such as cell cycle regulation, development, apoptosis, stress response, and tumourigenesis [2, 3]. Accordingly, miRNA alterations may contribute to many human diseases [4]. In fact, deregulated miRNA expression has been observed in various cancer types, such as multiple myeloma (MM) [5, 6]. miRNAs can act as both tumor suppressors and oncogenes, depending on the context and target genes [7, 8]. The regulatory mechanisms underlying miRNAs and their target mRNAs remain unclear: a single miRNA is capable of regulating >200 mRNAs, and a single mRNA may be regulated by multiple miRNAs [9]. Some studies have shown that miRNAs may not primarily act by repressing a few cancer-related genes but by disturbing a regulatory network in which these cancer-related genes play crucial functional roles [10, 11]. Thus, identification of context-dependent miRNA-mRNA modules is an important step to elucidate their synergistic effect on the pathogenesis of complex diseases.

Several computational methods have been previously developed for the discovery of miRNA-mRNA modules [12–18]. Early efforts primarily focused on computational predicted miRNA-mRNA pairs and detection of miRNA regulatory modules at the sequence level [16]. However, miRNA and mRNA expression were not taken into consideration. MiRNAs that are regulatory in one experimental scenario may not be regulatory in another [12]; expression information is essential for the identification of biologically meaningful miRNA-mRNA modules. Recently, integrated analysis of both sequence information and expression profiles of miRNAs and mRNAs was proposed to identify functional miRNA-mRNA regulatory modules [12–15, 19, 20]. Joung et al. [13, 14] discovered miRNA-mRNA modules using a combination of putative miRNA-mRNA pairs and expression data; however, correlations between the expression of miRNAs and mRNAs were not considered. Liu et al. [15] identified modules in two steps: (i) discovering the putative networks given the target information of miRNAs and mRNAs and (ii) deriving functional miRNA-mRNA regulatory modules on expression data given the putative networks. Considering that the computational predicted miRNA targets exhibit a high false positive discovery rate and that the targeting relationship between miRNAs and genes is far from complete, the first step that is based on target information exhibits an innate defect concerning the identification of modules. Jayaswal et al. [12] proposed an improved method: first, identification of miRNA and mRNA clusters using both target information and expression data; and second, estimation of the association between the two types of clusters to select potential regulatory miRNA-mRNA modules with statistically significant associations. However, this method was based on expression correlations under all available conditions rather than a subset of conditions, and the procedures for identification of miRNA and mRNA clusters were separated:; thus, it was limited to identification of miRNA-mRNA functional modules under the same specific conditions.

**Figure 1:**
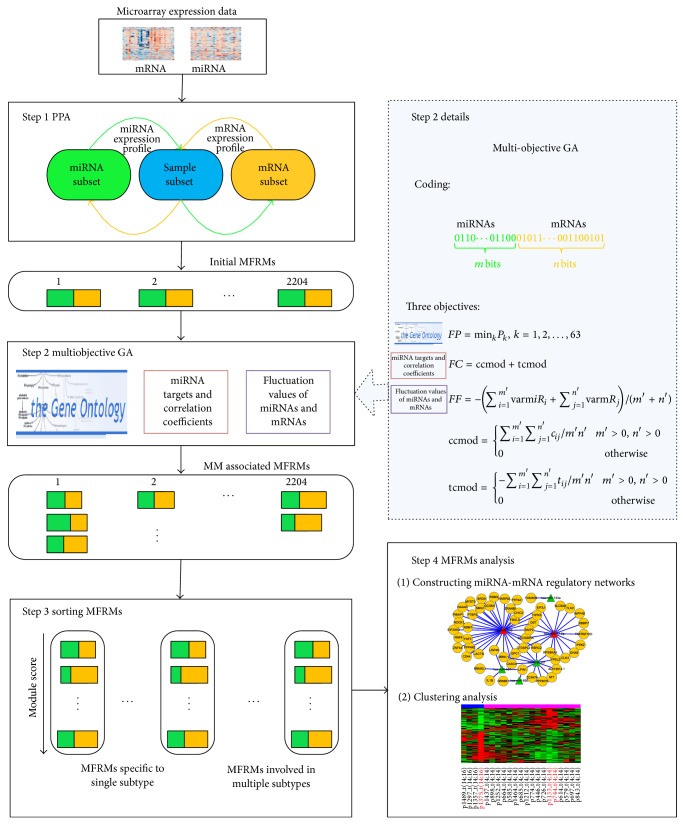
Workflow to identify MFRMs and MFRMs analysis.

For heterogeneous diseases, the miRNA-mRNA regulatory mechanism may be different in various subtypes, leading to differences in clinical behavior. In order to illustrate the pathogenesis of different subtypes, identification of context-dependent miRNA-mRNA functional regulatory modules (MFRMs) is important. In this study, we propose a novel method (Figure 1) for the genome-wide identification of MFRMs for different genetic subtypes of heterogeneous diseases. We applied the novel method on MM, which is characterized by significant heterogeneity at the molecular level [21] and divided into several subtypes on the basis of chromosomal abnormalities, such as t(4;14), t(14;16), t(11;14), and* RB* deletion [22]. We identified abundant subtype specific MFRMs associated with MM pathogenesis. The miRNA-mRNA regulatory networks were constructed based on MFRMs and provided numerous subtype specific miRNA-mRNA interactions. Clustering analysis showed that the MFRMs involved in multiple MM subtypes could separate the corresponding MM subtypes, indicating that these MFRMs could potentially aid in elucidation of the mechanisms underlying differences in clinical behavior.

## 2. Materials and Methods

### 2.1. Preparation of the Data Set

The matched expression profiles of miRNAs and mRNAs of MM were obtained from the studies of Gutiérrez et al. [23] (GSE16558). According to cytogenetic abnormalities, the 60 patients were classified into five subtypes: 17 patients with t(4;14); 11 with t(11;14); four with t(14;16); 15 with* RB* DEL (*RB* deletion as a unique abnormality,* RB* deletion, and P53 deletion); and 13 with NFISH (Normal Fish).

The seven target prediction data sources were obtained from DIANA-microT [24, 25], PicTar5 [26], RNA22 (R3/R5) [27], RNAhybrid [28], TargetScan [29, 30], and miRanda [31, 32]. The MM associated genes (see Table S1 in Supplementary Material available online at http://dx.doi.org/10.1155/2014/501262) were collected from three databases: Online Mendelian Inheritance in Man (OMIM), the Cancer Genome Project (CGP), and Genetic Association Database (GAD).

### 2.2. MFRMs Identification and Analysis

The methodology utilized in this study is illustrated in Figure 1. First, we identified initial comodules on matched mRNA and miRNA expression profiles using PPA [33]. A comodule is an ensemble of certain miRNAs, mRNAs, and samples, in which miRNAs and mRNAs exhibit similar patterns of expression across the same samples. The samples in the comodule imply the specific conditions under which the miRNAs and mRNAs act cooperatively. Second, to derive coherent modules associated with the pathogenesis of MM, we integrated GO BP [34] and miRNA target information to identify MFRMs in each comodule by multiobjective GA. Three optimization objectives were defined: (i) the minimum enriched *P* value on MM associated GO terms; (ii) the correlation coefficient and target coefficient (see Methods 2.3) of the module; and (iii) variations of expression values of miRNAs and mRNAs in the module. The multiobjective GA iteratively searched Pareto optimal solutions with three objectives and obtained noninferior MFRMs for each comodule. Next, we sorted the MFRMs according to their scores on three objectives. Finally, the top modules in the ranking results were identified and utilized to construct miRNA-mRNA regulatory networks or for clustering analysis.

### 2.3. Discovery of Comodules by PPA

We utilized the PPA [33] to identify comodules. The PPA is a modular analysis approach operating on two large-scale data sets that share one common dimension. Kutalik et al. [33] demonstrated that PPA could identify coherent patterns across paired data sets more effectively compared to classical approaches like clustering, regression, or SVD. A further advantage is that PPA provides context-dependent modules across paired data sets.

Let **E**_*N*_*G*_×*N*_*C*__ and **R**_*N*_*D*_×*N*_*C*__ represent paired gene expression data matrix and miRNA expression data matrix, respectively. *N*_*G*_, *N*_*D*_, and *N*_*C*_ represent the number of genes, miRNAs, and samples, respectively. Then the PPA is summarized in Pseudocode 1, where |**x**|, *μ*(**x**), and *σ*(**x**) denote the norm, mean, and standard deviation of the components *x*_*i*_ in the vector **x**; $\hat{x}=x/\left|x\right|$; *t*_*G*_, *t*_*D*_, and *t*_*C*_ denote the threshold of genes, miRNAs, and samples, respectively; **E**_*G*_ and **E**_*C*_ represent the gene expression matrix normalized across genes and samples, respectively; **R**_*D*_ and **R**_*C*_ represent the miRNA expression matrix normalized across miRNAs and samples, respectively.

Starting with the candidate set of genes (**g** ^(0)^), the mRNA expression profile (**E**_*N*_*G*_×*N*_*C*__) was used to identify samples (${\hat{c}}^{\left(n\right)}$) in which these genes were coexpressed. Next, the miRNA expression profile (**R**_*N*_*D*_×*N*_*C*__) was utilized to select miRNAs (${\hat{d}}^{\left(n\right)}$) that also exhibited a coherent expression in these samples (${\hat{c}}^{\left(n\right)}$). This set of miRNAs (${\hat{d}}^{\left(n\right)}$) was then utilized to refine the set of samples (${\hat{\tilde{c}}}^{\left(n\right)}$) by eliminating those which had an incoherent miRNA expression and adding others that behave similarly across these miRNAs. Finally, this refined set of samples (${\hat{\tilde{c}}}^{\left(n\right)}$) was used to probe for mRNAs (${\hat{g}}^{\left(n\right)}$) coexpressed in these samples. This alternating procedure was reiterated until it converged to stable sets of mRNAs, samples, and miRNAs: comodules.

### 2.4. Identification of MM Associated GO BP

To identify MM associated GO BP, we conducted cumulative hypergeometric distribution test to identify specific biological processes enriched with the MM associated genes. A total of 63 MM associated GO BP were identified (*P* < 0.05, Bonferroni corrected, Table S2).

### 2.5. Identification of MM Associated MFRMs Based on Multiobjective GA

To identify biologically meaningful coherent modules, we utilized a multiobjective genetic algorithm to extract MFRMs for each comodule. Let *m* be the number of miRNAs in a comodule and *n* be the number of mRNAs. Our aim is to extract a subset of miRNAs from the *m* miRNAs and a subset of mRNAs from the *n* mRNAs and construct a MFRM in which (i) the extracted subset of mRNAs is significantly enriched in the MM associated GO BP, (ii) miRNA expression exhibits a significant negative correlation with mRNA expression across the samples in the comodule, and, concurrently, the miRNAs and mRNAs exhibit a strong targeting relationship, and (iii) their expression values vary greatly among different subtypes. To this end, we defined three optimization objectives (i.e., the fitness function) as follows:

(1) $$\begin{array}{c} FP=\underset{k}{\min}\;P_{k},\;k=1,2,\ldots ,63,\\ FC=\text{ccmod}+\text{tcmod},\\ FF=-\frac{\left(\sum_{i=1}^{m^{\prime }}\text{varmi}R_{i}+\sum_{j=1}^{n^{\prime }}\text{varm}R_{j}\right)}{\left(m^{\prime }+n^{\prime }\right)}, \end{array}$$

where *P*_*k*_ was the *P* value (Bonferroni corrected) of the *k*th GO term enrichment on the subset of mRNAs. The first objective function *FP* represented the minimum *P* value of 63 MM associated GO term enrichments. The second objective function, *FC*, reflected the coherence of the module, where ccmod and tcmod were the correlation coefficient and target coefficient of the module, respectively:

(2) $$\begin{array}{c} \text{ccmod}=\left\{\begin{array}{cc} \overset{m^{\prime }}{\underset{i=1}{\sum }}\overset{n^{\prime }}{\underset{j=1}{\sum }}\frac{c_{ij}}{m^{\prime }n^{\prime }} & m^{\prime }>0,\;\;n^{\prime }>0\\ 0 & \text{otherwise}, \end{array}\right.\\ \text{tcmod}=\left\{\begin{array}{cc} -\overset{m^{\prime }}{\underset{i=1}{\sum }}\overset{n^{\prime }}{\underset{j=1}{\sum }}\frac{t_{ij}}{m^{\prime }n^{\prime }} & m^{\prime }>0,\;\;n^{\prime }>0\\ 0 & \text{otherwise}, \end{array}\right. \end{array}$$

where *m*′ and *n*′ were the number of selected miRNAs and mRNAs, respectively. The term *c*_*ij*_ represented the Pearson correlation coefficient of the *i*th miRNA and *j*th mRNA' expression value across the samples in the comodule (*P* value <0.05). The term *t*_*ij*_ represented the target coefficient of the *i*th miRNA and *j*th mRNA. The target coefficient between miRNA and mRNA was defined as the frequency that the mRNA was predicted as the target of the miRNA in seven target prediction data sources. The correlation coefficient ccmod and target coefficient tcmod of a module were defined as the average correlation coefficient and target coefficient of all miRNA-mRNA pairs in the module, respectively. The third objective function, *FF*, denoted the variations of miRNAs and mRNAs expression in the module, where varmi*R*_*i*_ and varm*R*_*j*_ were the between class variances of the *i*th miRNA's expression value and *j*th mRNA's expression value across five subtypes of MM, respectively. Both variances were normalized to between 0 and 1.

We used the “bit string” type to encode the individuals in the population *X*. Every individual *x* in *X* was encoded as a bit string with length *m* + *n*.

(3) $$\begin{array}{c} \underset{m\;\;\text{bits}}{\underset{︸}{0110\ldots 01100}}\underset{n\;\;\text{bits}}{\underset{︸}{01011\ldots 001100101}}. \end{array}$$

The first *m* bits represented *m* miRNAs in the comodule, and the remaining *n* bits represented *n* mRNAs in the comodule. The “0” represented the miRNA or mRNA selected into MFRM and “1” represented the miRNA or mRNA not selected. The number of “1” in the first *m* bits and remaining *n* bits was *m*′ and *n*′, respectively. The multiobjective optimization is formulated as

(4) min⁡x∈X  Fx=FPx,FCx,FFxTis.t.  FPx<0.05is.t.  m′,n′≥1,

where *FP*, *FC*, and *FF* are the objective functions defined as above. The solutions with fitness function *FP*(*x*) ≥ 0.05 were not kept for further investigation as the mRNAs lists of these solutions were not significantly enriched on any MM associated biological process.

### 2.6. Sorting MFRMs

MFRMs can be classified into six categories according to the condition under which they act: t(11;14), *RB* DEL, t(4;14), NFISH and t(14;16) subtype specific MFRMs, and heterogeneous MFRMs. The first five categories of MFRMs are specific to a single subtype, whereas the last category is involved in multiple subtypes (Figure 1). We sorted the MFRMs in each category separately. First, we sorted the MFRMs according to each objective and achieved the ranks *R*_1_, *R*_2_, and *R*_3_ on three objectives, respectively. The final score of a MFRM was then defined as the weighted sum of the three ranks:

(5) $$\begin{array}{c} S=\alpha R_{1}+\beta R_{2}+\gamma R_{3}. \end{array}$$

We set *α* = *β* = *γ* = 1/3. Finally, the MFRMs were sorted by their final scores in descending order.

## 3. Results

### 3.1. The Comodules Discovered by PPA

**Figure 2:**
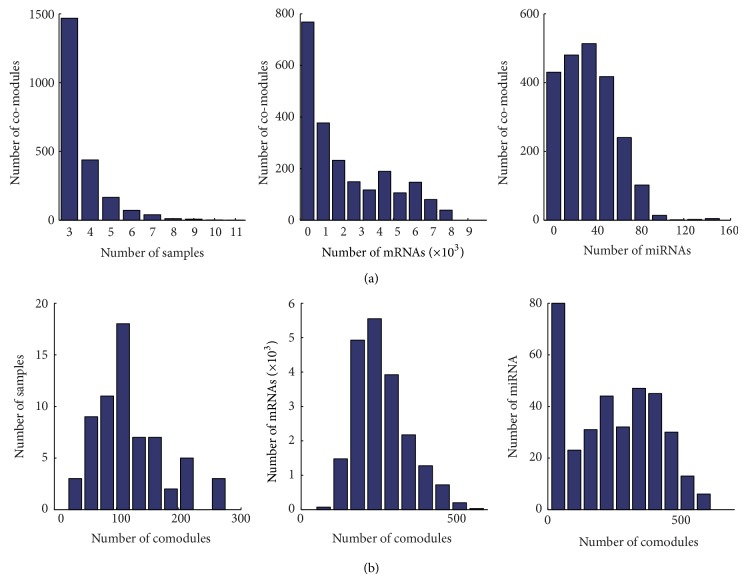
Comodule statistics. (a) The distribution of the number of comodules according to the number of samples, mRNAs, and miRNAs they contained. (b) The distribution of the number of samples, mRNAs, and miRNAs according to the number of comodules in which they were included.

We applied the PPA to the matched miRNA and mRNA expression profiles of MM and produced 2204 comodules which contains mRNAs, miRNAs, and samples. In each comodule, mRNAs and miRNAs exhibit coherent expression across the same samples. These samples imply the specific conditions under which the miRNA-mRNA module acts. For example, if the samples in a comodule all belong to subtype t(4;14), we refer to the miRNA-mRNA module as t(4;14) specific module. The miRNAs and mRNAs in the t(4;14) specific module are coexpressed only in t(4;14) samples but not in samples with other subtypes. Thus, the miRNAs and mRNAs in the t(4;14) specific module may exhibit a function specific to t(4;14). The miRNA-mRNA modules can be classified into six categories according to the condition under which they act, that is, t(11;14), *RB* DEL, t(4;14), NFISH and t(14;16) subtype specific modules, and heterogeneous modules (in other words, the samples in the corresponding comodule belong to different MM subtypes; Figure S1 shows a heterogeneous module). Among the 2204 comodules, we identified 14, 58, 41, 15, and two comodules specific to MM subtype t(11;14), *RB* DEL, t(4;14), and NFISH and t(14;16), respectively.  Figure 2 describes the distribution of the number of samples, mRNAs, and miRNAs attributed to 2204 comodules. The majority of comodules contained less than 2000 mRNAs and 60 miRNAs, and a few mRNAs and miRNAs acted as “hubs” by being part of up to 600 different comodules.

To assess the biological relevance of the mRNAs in the modules, we tested the functional homogeneity of the mRNAs in each module. A set of mRNAs is defined as functionally homogeneous if it is significantly enriched in at least one GO biological process category [34, 35]. Among the 2204 modules, 1679 (76.2%) were functionally homogeneous (*q*-value <0.05, FDR correction), indicating that the majority of modules discovered by PPA were biologically meaningful. Thus, the PPA was reliable to perform on matched miRNA and mRNA expression profiles and identify biologically meaningful miRNA-mRNA modules.

### 3.2. MFRMs Associated with MM Identified by Multiobjective GA

The miRNA-mRNA modules in the above section were identified only based on the expression correlation of miRNAs and mRNAs. To identify modules that are more biologically meaningful, there are still two important aspects need to be considered: the miRNA-target relationships and identification of modules that associated with the pathogenesis of given disease. To this end, we applied multiobjective GA on each comodule to extract MFRMs by integrating miRNA target information and MM associated GO BP (See Methods).

For each comodule, the multiobjective GA produced a Pareto optimal solution set of noninferior MFRMs. More significant expression correlations and stronger target relationships between the miRNAs and mRNAs were observed in the extracted MFRMs. For example, the multiobjective GA got four MFRMs on comodule 1680 (Table 1). Each MFRM was enriched on MM associated GO BP (*FP* < 0.05). Both the expression correlation coefficient of miRNAs and mRNAs and the target coefficient of the module were optimized. The second objective *FC* which reflected the expression correlation and target relationship was improved from −0.1599 in the original comodule to −0.1774, −0.2049 and −0.3456 in three functional modules, respectively. Although *FC* of the third MFRM was inferior, the variations of miRNAs and mRNAs expression (*FF*, the third objective) in this MFRM were the best. The larger the *FF*, the larger the variation of miRNA and mRNA expression among different MM subtypes and the more subtype specific the MFRM. Comodule 1680 contained four samples: p709, p831, p841 and p1204 which all belonged to subtype *RB* DEL. This indicated that the miRNAs and mRNAs in the MFRM were only co-expressed in samples with MM subtype *RB* DEL. Because the mRNAs in the MFRMs were significantly enriched on MM associated GO BP, the MFRMs extracted from comodule 1680 were *RB* DEL specific MFRMs and may represent a regulatory mechanism leading to the specific pathogenesis of subtype *RB* DEL. Similarly, we obtained subtype specific MFRMs for other MM subtypes, such as t(4;14), t(11;14), t(14;16), and NFISH. Multiobjective GA may produce more than one MFRM for each comodule. Figure S2 shows the distribution of the number of MFRMs extracted from each comodule. Most comodules produced no more than five MFRMs. The MFRMs were sorted according to the three objectives (see Methods) and those with the highest rank had priority for further investigation.

### 3.3. The miRNA-mRNA Regulatory Networks Provided a Modular Outlook at Subtype Specific miRNA-mRNA Interactions: Two Case Studies

We obtained abundant subtype specific MFRMs for each MM subtype. We focused on the MFRMs that ranked the highest, and then constructed miRNA-mRNA regulatory networks based on the expression correlation and target relationship between miRNAs and mRNAs in the MFRM. A miRNA-mRNA pair was connected with an edge if it concurrently satisfied two condition: (i) the miRNA exhibited a significant negative correlation with the mRNA across the samples in the comodule; and (ii) the mRNA was predicted as a target of the miRNA by at least one miRNA target prediction algorithm. Two cases are presented below: a *RB* DEL specific MFRM and a t(4;14) specific MFRM.

#### 3.3.1. *RB* DEL Specific MFRMs

**Figure 3:**
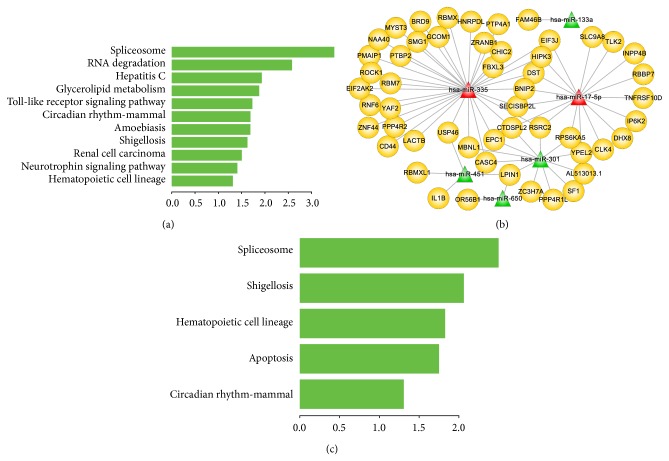
MiRNA-mRNA functional regulatory network of* RB* DEL specific MFRM 1680-1 and functions of miRNAs and mRNAs. (a) Significant biological pathways that genes of MFRM 1680-1 identified by multiobjective GA participated in. The *X*-axis represents −log⁡⁡10 transformation of *P* value. (b) miRNA-mRNA functional regulatory network of* RB* DEL specific MFRM 1680-1. Six miRNAs and 52 genes were involved in the network. miRNA and mRNA were connected with an edge if and only if the mRNA was targeted by the miRNA and there was a significant negative correlation between miRNA and mRNA expression. The miRNAs in red color were previously reported to play key roles in the pathogenesis of MM. (c) Significant biological pathways that genes of miRNA-mRNA functional regulatory network of* RB* DEL specific MFRM 1680-1 participated in. The *X*-axis represents −log⁡⁡10 transformation of *P* value.

*RB* DEL was a MM subtype that exhibited high morbidity rate, increased proliferative activity, and shorter overall survival [36]. We focused on the MFRM 1680-1 which ranked first among the *RB* DEL specific MFRMs. Firstly, we performed the functional enrichment analysis of mRNAs, indicating the functional roles of mRNAs belonged to this *RB* DEL specific MFRM. As shown in Figure 3(a), mRNAs in the MFRM significantly participated in several biological pathways that directly related with tumor. For example, Spliceosome is the most significant pathway; the study of Quidville et al. suggested that the deregulation of spliceosome induces mTOR Blockade and they provided the component of spliceosome as new therapeutic target of tumor [37]. Then, the miRNA-mRNA regulatory network was constructed based on the reverse expression and miRNA-target relationships (Figure 3(b)). There were 6 miRNAs and 52 genes in this MFRM (see Supplementary Materials for details). The functional enrichment analysis of these genes indicating that miRNAs and mRNAs in this MFRM significantly involved in the spliceosome and apoptosis biological pathways (Figure 3(c)), which are directly related to the occurrence and progression of tumor [37, 38]. Among these 6 miRNAs in MFRM, up to four miRNAs including miR-335, miR-17-5p, miR-451, and miR-301 were involved in a broad range of cancers [39, 40], such as acute lymphoblastic leukemia (ALL) [41], acute myeloid leukemia (AML) [42], and chronic myeloid leukemia (CML) [43]. In particular, miR-335 and miR-17-5p were connected with 33 and 17 mRNAs, respectively, thus exhibiting the important roles played in the network. Ronchetti et al. [44] reported that miR-335 was recurrently overexpressed in a fraction of primary tumors, possibly influencing plasma cell homing and/or interactions with the bone marrow microenvironment. miR-17-5p was a key regulator of the G1/S-phase cell cycle transition [45]. The study of Zhou et al. indicated that miR-17-5p exhibits a high expression level in myeloma cells and it may participate in the induction of p21Waf1/Cip1 expression, which relevant to the cell-cycle arrest process [46].* MYC* has been reported to play a causal role in the progression of monoclonal gammopathy to MM [47].* EPC1*, which interacts with miR-335 in the miRNA-mRNA regulatory network, has been shown to participate in growth regulation and has been suggested to be involved in a* MYC*-centered regulatory network [48]. CD44 was also directly connected with miR-335 and relevant to tumor. Purushothaman and Toole indicated CD44 serves as the binding partner of serglycin participate in the progression of MM [49]. Bjorklund et al. suggested that CD44 may contribute to the lenalidomide resistance in MM [50]. Moreover, miR-451 has previously been identified as one of the signatures capable of accurate discrimination of ALL from AML [41]. Because ALL, AML, CLL, MM, and lymphoma are all hematological malignancies, it is likely that these miRNAs also played special functional roles in MM. Of the 52 genes, many genes have been reportedly involved in various cancers, such as* HIPK3* [51],* RSRC2* [52],* BPAG1* [53], and* EPC1* [48]. Eight genes were annotated on apoptosis process, including* BNIP2*,* CD44*,* HIPK3*,* IP6K2*,* IL1B*,* PMAIP1*,* ROCK1*, and* TNFRSF10D*. They interacted with miR-335, miR-17-5p, and miR-451, further indicating the central role of these miRNAs in the regulatory network. This suggests that the miRNAs and mRNAs in the network worked together and contributed to the pathogenesis MM subtype Del* RB*.

#### 3.3.2. The t(4;14) Specific MFRMs

**Figure 4:**
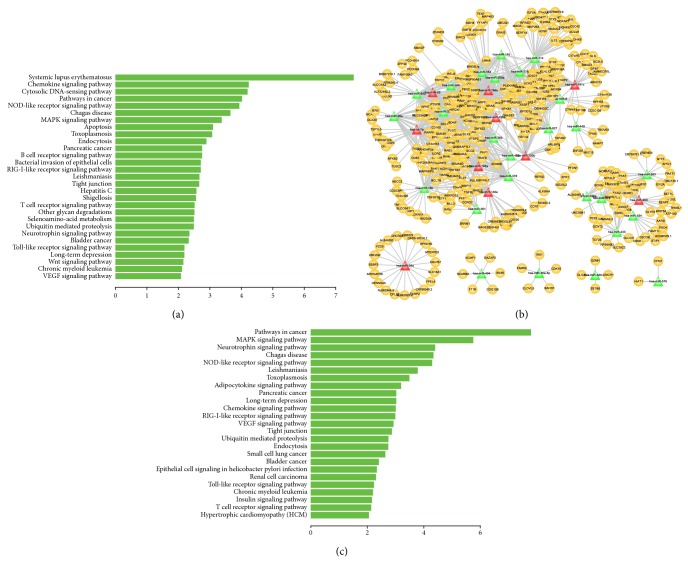
miRNA-mRNA functional regulatory network of t(4;14) specific MFRM 1121-2 and functions of miRNAs and mRNAs. (a) Significant biological pathways that genes of MFRM 1121-2 identified by multiobjective GA participated in. The *X*-axis represents −log⁡⁡10 transformation of *P* value. (b) miRNA-mRNA functional regulatory network of t(4;14) specific MFRM 1121-2. There were 36 miRNAs, 382 mRNAs and 983 edges involved in the network. The miRNAs in red color were previously reported to play key roles in the pathogenesis of MM. (c) Significant biological pathways that genes of miRNA-mRNA functional regulatory network of t(4;14) specific MFRM 1121-2 participated in. The *X*-axis represents −log⁡⁡10 transformation of *P* value.

MM subtype t(4;14), translocation of a region of chromosome 4 to chromosome 14, was highly associated with poor prognosis [54–57]. We firstly carried out functional enrichment analysis on mRNAs in MFRM 1121-2 which is the top ranked t(4;14) specific MFRMs. As a result, genes in the module significantly involved in many biological pathways such as “NOD-like receptor signaling pathway”, “MAPK signaling pathway”, “apoptosis,” and “tight junction” that have been known as hallmark processes of tumor (Figure 4(a)). Then, we constructed the miRNA-mRNA regulatory network of this t(4;14) specific MFRM, which including 36 miRNAs, 382 mRNAs and 983 edges (Figure 4(b)). Pathway enrichment analysis were also performed on these 382 mRMAs, the results suggest that miRNAs and genes in the MFRM were significantly involved in the biological pathways that directly related with tumor (Figure 4(c)). There were eight sub-networks identified, and most miRNAs and mRNAs were incorporated into the largest subnetwork. In the largest subnetwork, several miRNAs (let-7a, miR-125a, miR-193b, miR-25, and miR-181c) that acted as hubs in the network were previously reported to be associated with MM pathogenesis [6, 58]. Let-7a and mir-125a played important role in the t(4;14) regulatory network, in concordance with a previous study by Lionetti et al. [58]. They found that patients with t(4;14) exhibited specific overexpression of the miRNA cluster with let-7e, miR-125a, and miR-99b. Bakkus et al. also reported that Let-7a has a higher expression level in both the MM patients and cell lines [59]. Changes expression of miR-125a and let-7f which is in the same family of let-7a contributes to the myelomagenesis and are also relevant to overall prognosis [60]. Furthermore, the expression of miR-125b which is the same family member of miR-125a is associated with the chemotherapeutic-induced cell death in MM [61]. MiR-193b was a member of the miR-193b-365 cluster, which was previously identified as part of the unique miRNA signature in MM [62]. Mir-25 was a member of the oncogenic cluster miR-106b-25. Pichiorri et al. [6] determined that the oncogenic cluster miR-106b-25, miR-181a and miR-181b, which belonged to the same gene family with mir-181c, was a miRNA signature in the malignant transformation from MGUS to MM. Upregulation of miR-25 and miR-181-a/b and inactivation of miR-34, a central player in a smaller subnetwork, could negatively regulate the expression of the tumor suppressor gene* p53* [6, 63, 64], and contribute to MM progression. Alteration in miRNA expression (such as miR-34, miR-25, miR-181a/b, and miR-30d) during the progression from MGUS to newly diagnosed MM could be partially responsible for* p53* inactivation [5]. miR-25 is connected with 42 mRNAs. Many of these mRNAs were cotargeted by other miRNAs in the network, such as* RALA*,* BAK1*,* BMF*, and* JARID2*.* RALA* was targeted by 11 miRNAs. The product of* RALA* belonged to the oncogene* RAS* family of proteins and was involved in the MAPK/ERK signal transduction pathway which is the hallmark process of tumor. The study of Lim et al. indicated that activation of RALA play important role in the Ras-induced tumorigenesis [65].* BAK1* and* BMF* were targeted by four and six miRNAs, respectively.* JARID2* is an ortholog of the mouse* jumonji* gene that negatively regulates cell proliferation: it was targeted by nine miRNAs in the network, suggesting that it may also play an important role in human MM. Aside from these genes, 56.3% of genes in the network were targeted by multiple miRNAs, exhibiting the synergistic regulatory mechanism of miRNAs; miRNAs, along with genes, comprised the complex network specific to t(4;14).

### 3.4. Heterogeneous MFRMs Were Able to Separate Corresponding Subtypes

**Figure 5:**
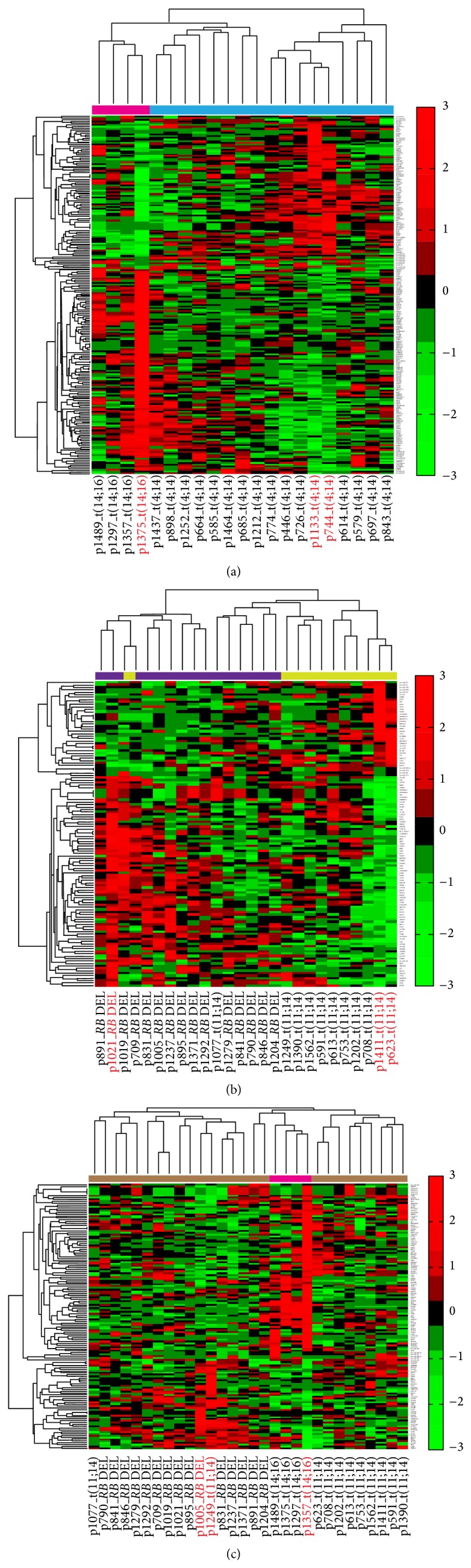
Hierarchical clustering diagrams. (a) The clustering diagram on t(14;16) and t(4;14) samples using the miRNAs and mRNAs in MFRM 1649-2. (b) The clustering diagram on* RB* DEL and t(11;14) samples using the miRNAs and mRNAs in MFRM 1653-3. (c) The clustering diagram on* RB* DEL, t(11;14) and t(14;16) samples using the miRNAs and mRNAs in MFRM 1962-4. Clustering could separate t(14;16) samples from other two subtypes. The samples in red color were from respective MFRMs. Although the MFRMs were identified in small subsets of samples only, they captured the principle characteristics of different MM subtypes.

Aside from MFRMs involved in a single subtype, we obtained a heterogeneous MFRM collection covering multiple MM subtypes. We found that some heterogeneous MFRMs exhibited differences in corresponding subtypes. For example, in heterogeneous comodule 1649 (Figure S1), expression of miRNAs and mRNAs between subtypes t(14;16) and t(4;14) was negatively correlated. The heterogeneous MFRMs extracted from this comodule potentially contain a mechanism that leads to a difference in subtype. We performed hierarchical clustering on both miRNAs and mRNAs in MFRM 1649-2 (ranked first among the MFRMs extracted from comodule 1649) for all t(4;14) and t(14;16) samples (Figure 5(a)). The clustering results confirmed that the 21 miRNAs and 196 mRNAs in this MFRM could separate t(4;14) from t(14;16) patients. Two miRNAs (hsa-let-7e; hsa-miR-125a) in this MFRM have been previously reported as miRNA signatures for their specific overexpression in t(4;14). Another miRNA miR-25, which was discussed above in t(4;14) specific MFRM 1121-2, was also incorporated in this MFRM. The 196 genes were significantly enriched in regulation of cell proliferation (*P* value = 6.9 × 10^−3^), regulation of ossification (*P* value = 8.2 × 10^−4^), blood vessel morphogenesis (*P* value = 3.7 × 10^−3^), blood vessel development (*P* value = 8.3 × 10^−3^), angiogenesis (*P* value = 1.3 × 10^−2^), and others, suggesting that the functional module contributes to the difference of t(4;14) and t(14;16). Next, we investigated the heterogeneous MFRM 1953-3, identified in patients with* RB* DEL and patients with t(11;14). Unsupervised hierarchical clustering showed that the 10 miRNAs and 115 genes could separate* RB* DEL from t(11;14), aside from one sample (Figure 5(b)). Interestingly, the heterogeneous MFRM 1962-4 acted in three subtypes:* RB* DEL, t(11;14), and t(14;16). The expression of miRNAs and genes was positively correlated between samples in* RB* DEL and t(11;14), but negatively correlated between samples in t(14;16) and* RB* DEL, t(11;14), suggesting that this module could lead to functional differences between t(14;16) and the other two subtypes. Clustering analysis using integrated miRNA and mRNA expression profiles showed that seven miRNAs and 138 mRNAs could separate t(14;16) from* RB* DEL and t(11;14) patients (Figure 5(c)).

### 3.5. The MFRMs Revealed Active miRNAs and mRNAs in Each MM Subtype

Overall, a few miRNAs and mRNAs act as “hubs” by being part of the majority of MFRMs. Further investigation of the miRNAs and mRNAs that appeared most frequently in subtype specific MFRMs will be helpful to elucidate the pathogenesis underlying each subtype. We referred to these miRNAs/mRNAs as subtype dependent active miRNAs/mRNAs. For subtype* RB* DEL,* CCDC50* was an active gene included in the majority of* RB* DEL specific MFRMs. It has been reported that tyrosine phosphorylation of* CCDC50* is important for inhibition of the* NFkB*-mediated apoptotic pathway [66] and* CCDC50* is required for survival in mantle cell lymphoma (MCL) and CLL cells [67].* KAT5* was another active gene: Zhao et al. [68] demonstrated that* KAT5* negatively modulated* c-Myb* transcriptional activity by recruiting histone deacetylases in human hematopoietic cells. Other active genes, like* NFKBIB*,* PIK3CA*,* RELA*,* LYN*, and* MAP2K7*, were involved in B cell and T cell receptor signaling pathways. These genes frequently appeared in* RB* DEL specific MFRMs, demonstrating that they played critical roles in* RB* DEL. The top 15 miRNAs and 50 mRNAs frequently included in each type of subtype specific MFRMs are listed in Table S3.

## 4. Discussion

Identification of subtype specific miRNA-mRNA modules is important for the study of heterogeneous diseases. Several points need to be considered regarding miRNA-mRNA modules: (i) the mRNAs are targeted by miRNAs in the same module; (ii) there may be a significant expression correlation of miRNAs and mRNAs; (iii) the functions that the miRNA-mRNA modules perform; (iv) the conditions under which the modules work. Most methods [12–16, 19] considered (i) and (ii) but ignored (iii) and (iv). Besides, the methods currently employed assign a miRNA/mRNA to only one module. However, a miRNA/mRNA may participate in different biological processes working with different genes and miRNAs. In this study, we used the PPA algorithm to identify miRNA-mRNA modules. The advantage of PPA is twofold. First, it can assign miRNAs and genes to multiple modules, which is well motivated from the biological point of view as the same gene can function in multiple processes under different conditions. Second, the PPA could identify context-dependent modules in which the miRNAs and genes are coexpressed in a subset of samples. These modules are widely ignored by many other clustering algorithms which calculate correlations over all samples. Another improvement we propose is the ability to identify condition-related MFRMs associated with predefined biological processes (e.g., MM associated GO BP). This process utilizes an integrated analysis of three pieces of biological data: GO BP, miRNA target information, and expression data based on multiobjective GA. The first objective *FP* utilizes the predefined MM associated GO BP to optimize the MFRMs. It ensures that the MFRMs are biologically meaningful and associated with the pathogenesis of MM. The second objective *FC* integrates both expression profiles and miRNA target information. It guarantees that the miRNA expression is significantly negatively correlated with the mRNA expression in the MFRM, and concurrently the miRNAs and mRNAs exhibit a strong targeting relationship. The last objective *FF* is based on the intuition that the expression values of miRNAs and genes which lead to pathogenesis and heterogeneity of MM may vary greatly among the different subtypes. Our method captures a resource of subtype specific MFRMs that constitute various specific functional mechanisms in each MM subtype that may lead to differences in clinical behavior.

**Figure 6:**
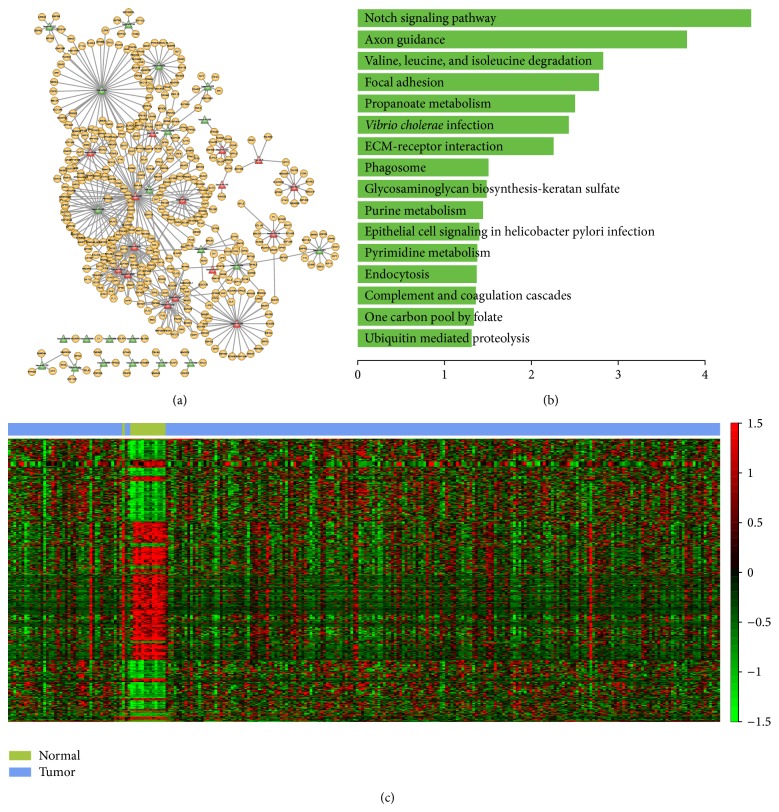
miRNA-mRNA functional regulatory network of breast cancer and functions of miRNAs and mRNAs. (a) miRNA-mRNA functional regulatory network. The miRNAs in red color were reported to relevant with breast cancer in miR2Disease database. (b) Significant biological pathways that genes of miRNA-mRNA functional regulatory network of breast cancer participated in. The *X*-axis represents −log⁡⁡10 transformation of *P* value. (c) The hierarchical clustering diagram on breast cancer dataset using the 38 miRNAs and 418 genes in MFRM.

In order to examine the robustness and extensive application of our method, we performed it on breast cancer data set, which is RNA-seq data of TCGA including 14 normal samples and 248 cancer samples (http://cancergenome.nih.gov/). In total, PPA algorithm obtained 66 modules, 4 of these modules (modules 1, 12, 27, and 42) were normal samples specific indicating the dysregulation of these modules were associated with breast cancer. We then constructed the miRNA-mRNA regulatory network and carried out functional analysis of module 1. The module 1 regulatory network contained 38 miRNAs, 418 genes, and 537 edges (Figure 6(a)). These miRNAs and genes were involved in several biological pathways that directly associated with tumor such as “focal adhesion”, “notch signaling pathway”, “purine metabolism,” and “ECM-receptor interaction” (Figure 6(b)). Of these 38 miRNAs in the network, up to 16 miRNAs were recoded to be relevant with breast cancer in miR2Disease database [69]. For example, the study of Guttilla and White indicate that the coordinately regulation of FOXO1 by miR-96 and miR-182 which involved in the miRNA-mRNA regulatory network was associated with the oncogenic state in breast cancer cells [70]. Furthermore, regulation of Rac1 signaling by ARF1 which directly interact with miR-96 in the regulatory network is associated with invasive breast cancer cells [71]. We used two-dimensional hierarchical clustering analysis to visualize the expression pattern of miRNAs and genes in the MFRM. As shown in Figure 6(c), these miRNAs and genes exhibit different expression pattern in normal and tumor samples. In summary, these results suggest that our method can robustly capture important MFRMs relevant to diseases when applied to RNA-Seq data.

We identified a large number of subtype specific MFRMs, such as MFRM 1680-1, 1121-2. The regulatory networks built on these two MFRMs were specific to* RB* DEL and t(4;14), respectively. The links in the regulatory networks predicted new potential subtype dependent miRNA-mRNA interactions. The genes in the two regulatory networks were significantly enriched in MM associated biological processes, such as apoptosis, and regulation of cell death. Although the miRNAs, genes and the regulatory mechanisms were different, they all contributed to the pathogenesis of their respective subtypes. Further investigation of other subtype specific MFRMs may uncover a different pathogenesis in each subtype.

For heterogeneous MFRMs involved in multiple subtypes, the miRNAs and mRNAs acted in different ways between the subtypes. For example, in MFRM 1649-2, approximately one third of mRNAs were upregulated in t(4;14) but downregulated in t(14;16). Clustering on three heterogeneous MFRMs showed that these MFRMs could separate different subtypes, although this only involved a small subset of the corresponding subtypes; the reason for this may be that all of the samples of the corresponding subtypes were not covered due, in part, to individual differences. Clustering results indicated that heterogeneous MFRMs captured natural differences and led to different subtypes. These MFRMs could potentially be helpful for identifying functional biomarkers of MM subtypes.

## Supplementary Material

Figure S1. Schematic representation of a co-module. The expression values of miRNAs and mRNAs were indicated by a color code ranging from green (under-expressed) to red (over-expressed). Note that in this co-module, two samples of t(4;14) exhibited similar profiles, whereas the sample of t(14;16) exhibited an inverse profile.
Figure S2. Distribution of the number of MFRMs extracted from each co-module.
Table S1. 72 MM associated genes from OMIM, CGP and GAD.
Table S2. 63 MM associated GO biological processes.
Table S3. The top 15 miRNAs and 50 mRNAs frequently included in each kind of subtype specific MFRM.

## Figures and Tables

**Pseudocode 1:**
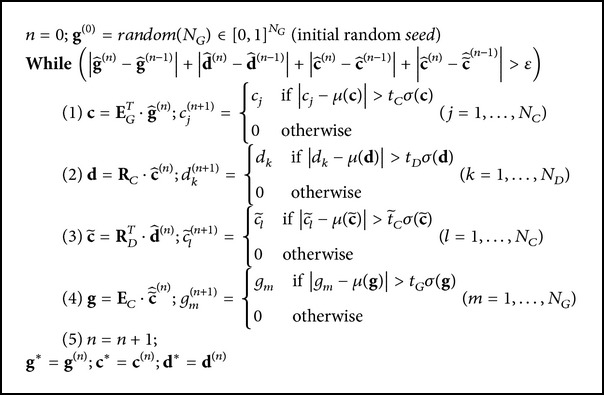


**Table 1:** Multiobjective GA on comodule 1860.

	Comodule 1680	MFRM 1860-1	MFRM 1860-2	MFRM 1860-3	MFRM 1860-4
Number of miRNAs	20	14	15	16	3
Number of mRNAs	511	224	347	209	283
*FP *	None^a^	0	0.031	0.024	0.020
*FC *	−0.1599	−0.1774	−0.2049	−0.1442	−0.3456
*FF *	−0.0469	−0.0610	−0.0548	−0.0665	−0.0339

^a^The mRNAs were not enriched on any MM associated biological process.
