# Supplementary figures and images for: Identification of Subtype Specific miRNA-mRNA Functional Regulatory Modules in Matched miRNA-mRNA Expression Data: Multiple Myeloma as a Case
Source: Biomed Res Int. 2015 Mar 19;2015:501262. doi: 10.1155/2015/501262 (PMC4385567; doi:10.1155/2015/501262)

p1375-t(14;16)

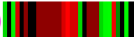

p1133-t(4;14)

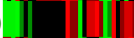

p744-t(4;14)

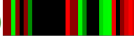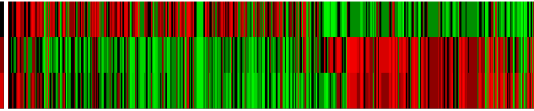

Three samples

32 miRNAs

464 mRNAs

Supplement: Supplementary file 1 — Figure S1. Schematic representation of a co-module. The expression values of miRNAs and mRNAs were indicated by a color code ranging from green (under-expressed) to red (over-expressed). Note that in this co-module, two samples of t(4;14) exhibited similar profiles, whereas the sample of t(14;16) exhibited an inverse profile. Figure S2. Distribution of the number of MFRMs extracted from each co-module. Table S1. 72 MM associated genes from OMIM, CGP and GAD. Table S2. 63 MM associated GO biological processes. Table S3. The top 15 miRNAs and 50 mRNAs frequently included in each kind of subtype specific MFRM. [file 501262.f1.zip › 501262.f1/Figure S1.pdf]

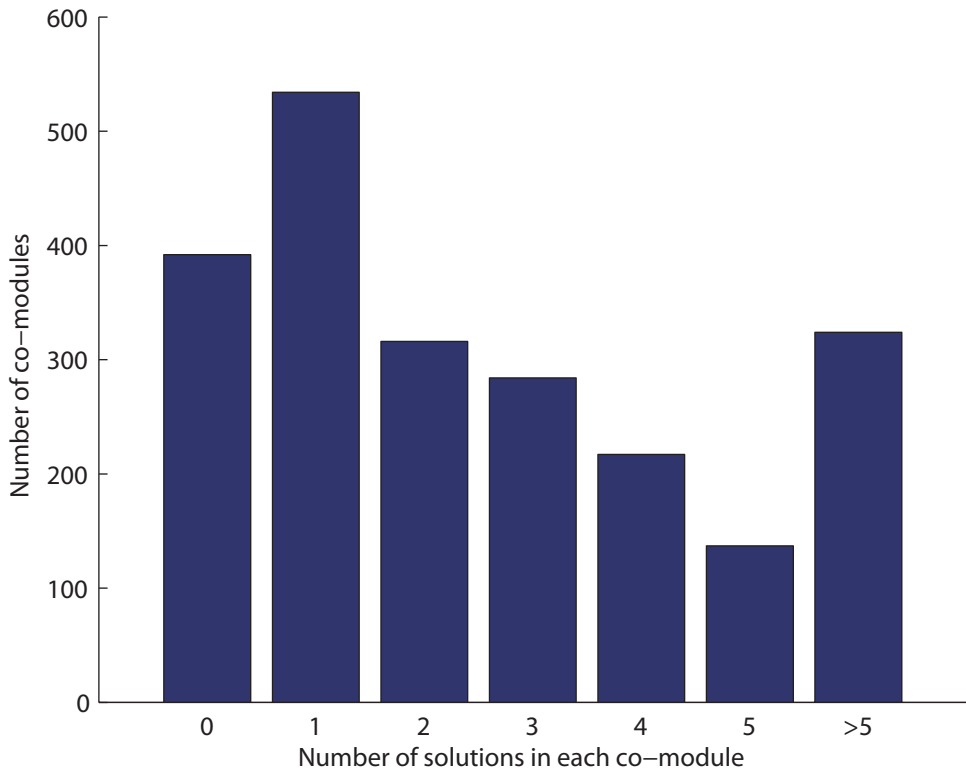

Supplement: Supplementary file 1 — Figure S1. Schematic representation of a co-module. The expression values of miRNAs and mRNAs were indicated by a color code ranging from green (under-expressed) to red (over-expressed). Note that in this co-module, two samples of t(4;14) exhibited similar profiles, whereas the sample of t(14;16) exhibited an inverse profile. Figure S2. Distribution of the number of MFRMs extracted from each co-module. Table S1. 72 MM associated genes from OMIM, CGP and GAD. Table S2. 63 MM associated GO biological processes. Table S3. The top 15 miRNAs and 50 mRNAs frequently included in each kind of subtype specific MFRM. [file 501262.f1.zip › 501262.f1/Figure S2.pdf]
